# P2X7 Receptor-Related Genetic Mouse Models – Tools for Translational Research in Psychiatry

**DOI:** 10.3389/fncir.2022.876304

**Published:** 2022-03-29

**Authors:** Lidia Urbina-Treviño, Iven-Alex von Mücke-Heim, Jan M. Deussing

**Affiliations:** ^1^Max Planck Institute of Psychiatry, Molecular Neurogenetics, Munich, Germany; ^2^Graduate School of Systemic Neurosciences, Ludwig Maximilian University of Munich, Munich, Germany; ^3^International Max Planck Research School for Translational Psychiatry, Munich, Germany

**Keywords:** P2X7 receptor, purinergic signaling, genetic mouse model, translational psychiatry, depression

## Abstract

Depression is a common psychiatric disorder and the leading cause of disability worldwide. Although treatments are available, only about 60% of treated patients experience a significant improvement in disease symptoms. Numerous clinical and rodent studies have identified the purinergic P2X7 receptor (P2X7R) as one of the genetic factors potentially contributing to the disease risk. In this respect, genetically engineered mouse models targeting the P2X7R have become increasingly important in studying designated immunological features and subtypes of depression *in vivo*. This review provides an overview of the P2X7R -related mouse lines currently available for translational psychiatric research and discusses their strengths, weaknesses, and potentials.

## Introduction

Depression is a common psychiatric disorder with a lifetime prevalence of 10.8% worldwide (James et al., 2018; Lim et al., 2018). Within the last few decades, the incidence and prevalence of depression have increased (Liu et al., 2020; Steffen et al., 2020b). This is alarming, since depression is highly associated with somatic and mental comorbidity, mortality, socioeconomic costs, and limited therapeutic reliability (Kessler, 2012; Gilman et al., 2017; Machado et al., 2018; Cuijpers et al., 2020; Steffen et al., 2020a; Sontheimer et al., 2021). Currently, depression is considered the main cause of disability worldwide [Friedrich, 2017; World Health Organizaton [WHO], 2017].

Recently, the concept of depression has been challenged, and different subtypes have been identified on the grounds of imaging, genetic, phenotypic, and immunometabolic factors (Musil et al., 2018; Tokuda et al., 2018; Milaneschi et al., 2020; Buch and Liston, 2021; de Kluiver et al., 2021; Kappelmann et al., 2021; Nguyen et al., 2021). In line with the immunometabolic hypothesis, studies have shown that genetic polymorphisms in inflammatory genes such as interleukin 1 beta (IL-1β), tumor necrosis factor alpha (TNFα) and C-reactive protein, influence depression incidence, severity, and treatment response (Barnes et al., 2017; de Kluiver et al., 2019, 2021; Draganov et al., 2019; Kappelmann et al., 2021). Meanwhile, psychosocial stress, the main environmental risk factor for depression, was linked to changes in adenosine triphosphate (ATP) mediated P2X7 receptor (P2X7R) signaling and related to neuroinflammation (Kendler et al., 1999; Iwata et al., 2013; Rohleder, 2014; Calcia et al., 2016; Nelson et al., 2017; Maydych, 2019; Ribeiro et al., 2019; Kim et al., 2020). Therefore, innate, and adaptive immunity involving P2X7R is currently deemed a key player in stress-induced depression (Wohleb et al., 2016; Leday et al., 2018; Giollabhui et al., 2020; Illes et al., 2020; Troubat et al., 2020; von Muecke-Heim et al., 2021). However, the role of P2X7R in this disorder still needs to be fully unraveled (Medina-Rodriguez et al., 2018).

In the past three decades, P2X7R -related genetically engineered mouse models have become increasingly relevant in the study of the connection between P2X7R signaling and depression *in vivo*. In this review, we briefly outline the general properties of the P2X7R and provide a comprehensive overview of currently available transgenic mouse lines.

## General Characteristics of P2X7R

Purinergic signaling is an evolutionarily ancient and ubiquitous cell-to-cell communication, which is involved in tissue homeostasis and multiple pathophysiological conditions, including mood disorders (Verkhratsky and Burnstock, 2014; Ribeiro et al., 2019; Bartoli et al., 2020). Although ATP is well known for its role as an energy carrier in cell metabolism, as well as a relevant cell-to-cell signaling molecule in diverse cell types under physiological conditions, it additionally acts as an extracellular messenger in the context of cell trauma (Burnstock, 2006).

The P2X7R belongs to the P2X family (P2X1-7), which comprises trimeric ligand-gated ion channels responsive to extracellular ATP (Di Virgilio et al., 2017). In the central nervous system (CNS), P2X7R is mainly expressed in microglia, astrocytes, and oligodendrocytes as well as in other immune cells (Jacobson and Müller, 2016; Adinolfi et al., 2018). Each P2X7R monomer has two transmembrane domains with intracellular C- and N-termini and a large ectodomain, where the ATP-binding sites are found at the interface between the monomers (Hattori and Gouaux, 2012; Karasawa and Kawate, 2016; McCarthy et al., 2019).

Once activated, P2X7R adopts an open conformation, allowing K^+^ efflux and Na^+^ and Ca^2+^ influx (Habermacher et al., 2016; Di Virgilio et al., 2018), triggering the assembly of the intracellular NLR family pyrin domain containing 3 (NLRP3) inflammasome and subsequent activation of caspase-1. This stimulates cell metabolism *via* glycolysis and upregulation of oxidative phosphorylation and causes IL-1β, IL-6, TNFα, and IL-18 release, eliciting a neuroinflammatory response (Di Virgilio et al., 2017; von Muecke-Heim et al., 2021). P2X7R activation also potentiates the innate immune response by inducing proliferation, recruitment and activation of microglia, macrophages, and lymphocytes (Dubyak, 2012; Monif et al., 2016; Colonna and Butovsky, 2017).

The P2X7R requires much higher concentrations of extracellular ATP [half maximal effective concentration (EC_50_) for ATP: 2–4 mM] than other members of the P2X family (EC_50_ range: 0.1–10 μM) (Khakh and North, 2012; Jacobson and Müller, 2016). This high activation threshold and its relatively slow desensitization make P2X7R a particularly relevant molecule in chronic inflammatory conditions (Khadra et al., 2013; Adinolfi et al., 2018; Andrejew et al., 2020; Calzaferri et al., 2020).

## Comparing Human and Murine P2X7R Properties

Human and mouse P2X7R s possess species-specific characteristics. The human receptor has a higher affinity for BzATP and ATP (EC_50_: 20 μM and 100 μM, respectively), compared to the murine receptor (EC_50_: 295 μM and 850 μM, respectively) (Moore and MacKenzie, 2008; Khakh and North, 2012). Similarly, the binding affinity for various pharmacological agents varies between different mammalian P2X7R s (Donnelly-Roberts et al., 2009). Furthermore, the human P2X7R has a comparatively higher deactivation speed, which likely depends on the C-terminal region of the receptor (Bartlett et al., 2014). Both human and murine P2X7R s undergo post-translational modifications, including N-linked glycosylation and palmitoylation. Due to the lack of specific ADP-ribosyl transferases, only the murine P2X7R is subject to ADP ribosylation at Arg^125^, which is located in close vicinity to the ATP binding region. This process leads to the ATP-independent activation of the receptor in the presence of NAD^+^ (Sluyter, 2017).

The genes encoding the murine and the human receptors are localized on chromosome 5 and 12, respectively, in a region of conserved synteny. The P2RX7 gene undergoes species-specific alternative splicing. To date, 13 human (P2X7R -A to K, P2X7-V3 and nf P2X7) and five mouse (P2X7R -A, B, C, D, and K) splice variants have been described, with P2X7R -A being the main functional variant in both species (Sluyter and Stokes, 2011; Pegoraro et al., 2021). Studies in mice have shown that some of these alternative variants, P2X7R -K and P2X7R -C in particular, can produce functional receptors that might play a specific role in processes such as cell growth, cell death and inflammation (Cheewatrakoolpong et al., 2005; Feng et al., 2006; Adinolfi et al., 2010; Stokes et al., 2010; Bartlett et al., 2014).

## P2X7R and Psychiatric Disorders

In genetic studies, single nucleotide polymorphisms (SNPs) in the *P2RX7* gene haven been linked to brain disorders such as depression, Alzheimer’s disease, or bipolar disorder (Lucae et al., 2006; Czamara et al., 2018; Andrejew et al., 2020). In particular, the region of chromosome 12 harboring the P2RX7 gene (12q24) has been linked to psychiatric disorders (Degn et al., 2001; Abkevich et al., 2003; Curtis et al., 2003; McGuffin et al., 2005; Shink et al., 2005). Foremost the SNP rs2230912, which causes the non-synonymous 1405 A > G transition and translates to a Gln460Arg substitution in the C-terminal intracellular domain of the protein (Barden et al., 2006; Lucae et al., 2006; Andrejew et al., 2020). Although the association between rs2230912 and mood disorders is inconsistent (Green et al., 2009; Grigoroiu-Serbanescu et al., 2009; Viikki et al., 2011), this SNP seems to influence symptom severity of mood disorders. Patients carrying the G allele experience longer disease course and greater symptom severity (Nagy et al., 2008; Hejjas et al., 2009; Soronen et al., 2011). Moreover, carriers of both alleles show subtle alterations in sleep patterns, suggesting a heterozygote disadvantage (Metzger et al., 2017b). Although a meta-analysis from 2014 did not detect an association between rs2230912 and mood disorders (Feng et al., 2014), a more recent meta-analysis, which included more studies, presented a significant association of rs2230912 with mood disorders (Czamara et al., 2018). However, a genome-wide association study, which identified risk variants for depression and bipolar disorder with genome-wide significance, did not detect any association related with the P2RX7 gene (Wray et al., 2018; Stahl et al., 2019).

The lack of consistency among studies may be explained by the fact that depression is a heterogeneous and polygenic disorder where many low-impact loci interact with each other and the environment to promote disease development (Peterson et al., 2018). Thus, studying the impact of a single SNP is insufficient to understand the complexity of depression genetics. Haplotype studies go one step further and consider combinations of polymorphisms found in the same region. Specifically, rs2230912 and rs1718119 (Ala348Thr) lead to a gain-of-function variant of P2X7R. In fact, carrying these two SNPs causes an increased production of IL-1β in response to ATP (Stokes et al., 2010) and a higher severity of depression (Vereczkei et al., 2019).

Translational research in rodents has shown that chronic stress causes extracellular ATP increase and P2X7R activation in the brain, which results in depressive-like behavior and impaired neuroplasticity (Iwata et al., 2016; Wohleb et al., 2016; Metzger et al., 2017b; von Muecke-Heim et al., 2021). Vice versa, blockage along the P2X7R -NLRP3-IL-1β cascade has been shown to promote stress resilience with microglia and monocytes playing an important part (Schiweck et al., 2020; von Muecke-Heim et al., 2021). These studies point out P2X7R’s signaling role as an important interface between chronic stress and the behavioral features of clinical depression.

In summary, a multitude of clinical and translational studies provide strong evidence that P2X7R may contribute to depression genesis, severity, and treatment response. This highlights the needed to further study the properties and functions of P2X7R in the context of stress-related disorders including depression.

## Genetic Mouse Models Targeting P2X7R

To elucidate the role of P2X7R signaling in physiological and pathological conditions *in vivo*, several loss- and gain-of-function as well as reporter mouse lines have been generated in the past few decades.

### Constitutive P2X7R Knockout Mouse Lines

**FIGURE 1:**
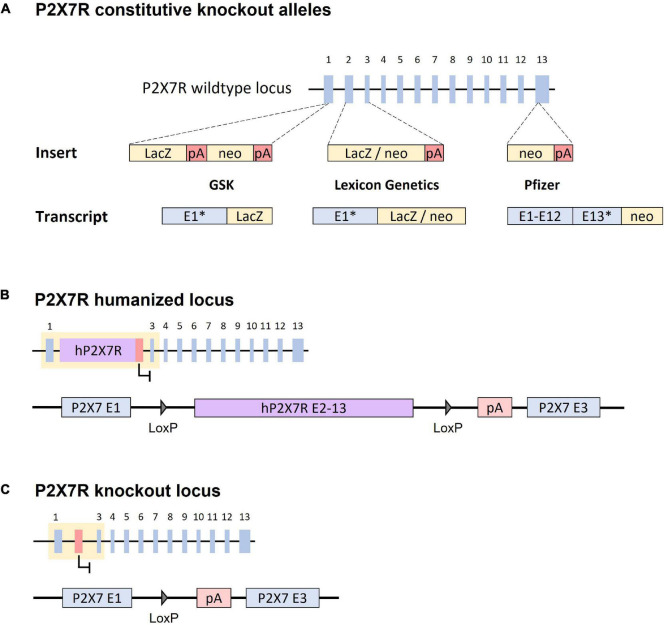
Strategies targeting the *P2RX7* gene in constitutive and conditional knockout mouse lines. **(A)** P2X7R constitutive knockout alleles. GSK (P2rx7*^tm1Ipch^*): a LacZ-neomycin (neo) cassette was inserted in exon 1, which results in an exon 1-LacZ fusion transcript. Lexicon Genetics (P2rx7*^tm1Lex^*): a LacZ-neomycin cassette was used to substitute exons 2 and 3, which results in an exon 1-LacZ-neo fusion transcript. Pfizer (P2rx7*^tm1Gab^*): a neomycin cassette was inserted in exon 13, which truncates the transcript in the C-terminal region. “*”: truncated P2X7R exon. Dashed lines indicated the insertion site of the construct. **(B)** Humanized P2X7R locus. The human cDNA sequence from exon 2–13 substitute’s murine exon 2. The cDNA sequence is flanked by LoxP sites. **(C)** P2X7R conditional knockout locus. Upon Cre recombinase activity, the cDNA fragment is deleted resulting in a disrupted P2RX7 gene. The loss of exon 2 prevents the expression of any functional P2X7R variants. “pA/red box”: Polyadenylation signal.

Three knockout mouse lines were created by pharmaceutical companies to investigate the consequences of a lack of P2X7R expression. The first P2X7R knockout mouse line was established by GlaxoSmithKline (GSK) by inserting a LacZ-neomycin reporter cassette into exon 1, which results in a fusion transcript comprising the 5′part of exon 1 and LacZ (Figure 1A; Sikora et al., 1999). A second knockout mouse line, generated by Pfizer Inc., (Pfizer), contains a neomycin selection cassette disrupting exon 13, which corresponds to the C-terminal region of the protein (Figure 1A; Solle et al., 2001). Finally, the knockout mouse line established by Lexicon Genetics involves as substitution of exons 2 and 3 by a LacZ-neomycin cassette, producing a fusion transcript by splicing exon 1 to the LacZ-neomycin construct (Figure 1A; Basso et al., 2009). In the initial studies, the authors analyzed P2X7R mRNA and protein expression and confirmed that P2X7R -dependent release of IL-1β and its pore forming function were impaired *in vitro* and *in vivo*. These studies also observed inflammatory hyposensitivity in the knockout animals (Csölle et al., 2013a). Therefore, the functional effects in these loss-of-function mouse lines are consistent with the lack of P2X7R expression.

Studies on constitutive P2X7R knockout mice generally show that genetic inactivation, similar to pharmacological inhibition of P2X7R, leads to a decrease of depressive-like behaviors under baseline (Basso et al., 2009; Csölle et al., 2013a) and stress conditions (Boucher et al., 2011; Csölle et al., 2013b; Yue et al., 2017). Although some studies could not detect an influence of P2X7R inactivation on anxiety-like phenotypes (Basso et al., 2009; Csölle et al., 2013a), others have reported reduced anxiety-like behaviors in P2X7R knockout mice even under baseline conditions (Boucher et al., 2011; Yue et al., 2017). However, it is important to note that further studies on these knockout animals have unveiled that P2X7R splice variants, some of which present residual or even altered P2X7R activity, are able to escape gene inactivation in the Pfizer and GSK mice (Nicke et al., 2009; Masin et al., 2012). Therefore, these lines cannot be considered fully deleterious for P2X7R, implying that conclusions from the studies that employed these mice should be drawn with care.

Besides classical targeting strategies, approaches involving transgenic short hairpin RNA-based knock-down (Delic et al., 2008) and CRISPR/Cas9-mediated gene deletion have also been used to disrupt P2X7R expression and function (Gao et al., 2018).

### Humanized P2X7R Mouse Lines

Although constitutive knockout mice provide valuable insights with respect to the physiology of the murine P2X7R, studying the human receptor in mice would allow for additional insights, for example with respect to the receptor’s *in vivo* pharmacology. To this end, a humanized P2X7R mouse model was generated. The humanized allele [P2rx7*^tm1.1(P2RX7)Jde^*] involves the knock-in of the human P2X7R cDNA (exons 2–13) downstream of exon 1 substituting murine exon 2 (Figure 1B). This way, the humanized P2X7R presents the same expression pattern as the endogenous murine protein in wild-type mice. Consistent with the previously described EC_50_ values of the human and murine receptors (Moore and MacKenzie, 2008; Khakh and North, 2012), the humanized P2X7R showed a 10-fold higher sensitivity to BzATP compared to its murine counterpart. The same strategy was used to generate another humanized mouse line harboring the Q460R polymorphism that had previously been linked to depression [P2rx7^tm2.1(P2RX7*)Jde^]. Studies on these humanized mouse lines revealed how a specific genetic alteration in the human protein interacting with environmental risk factors can impact depression- and anxiety-related behavior. Similar to human heterozygous subjects, heterozygous mice suffered from altered sleep architecture and quality. These findings might reflect a prodromal disease state, which translates into higher stress vulnerability under conditions of chronic social stress (Metzger et al., 2017b).

### Conditional P2X7R Mouse Lines

Another trait of the humanized P2X7R line is its susceptibility to conditional inactivation. The human cDNA fragment is flanked by loxP sites and thus can be removed by Cre-mediated recombination (Figure 1B). This results in a truncated murine gene, which lacks the ability to produce a functional receptor (Figure 1C). Therefore, crossing these mice with respective Cre driver mouse lines enables spatially and temporally controlled P2X7R inactivation. In contrast to constitutive knockout mice, this approach prevents potential developmental or pleiotropic effects of P2X7R inactivation due to its early and relatively broad expression, which naturally entails compensatory mechanisms. The lack of P2X7R following Cre-mediated inactivation was tested functionally, by assessing the capability of peritoneal macrophages to produce IL-1β, as well as lack of Ca^2+^ influx after stimulation with BzATP. The deficiency of the known P2X7R variants was also tested demonstrating the absence of any functional transcripts (Metzger et al., 2017a).

In addition, a conditional allele flanking murine exon 2 with loxP sites has been generated by the European Conditional Mouse Mutagenesis (EUCOMM) Program [P2rx7*^tm1a(EUCOMM)Wtsi^*]. It has been demonstrated that Cre-mediated deletion of exon 2 results in a complete loss of P2X7R function (Kaczmarek-Hajek et al., 2018; Douguet et al., 2021).

### P2X7R Reporter Mouse Lines

There has been a significant advance in the biological targeting of P2X7R, with the recent development of a specific P2X7R nanobody (Kaczmarek-Hajek et al., 2018). However, commercially available P2X7R antibodies still show considerable deficits in functionality and specificity (Anderson and Nedergaard, 2006; Illes et al., 2017). In general, the comparably low expression contributes to the challenge of reliably detecting P2X7R expression in the CNS. Therefore, mouse lines that express fluorescent reporters under the control of the P2X7R promoter or a tagged P2X7R represent valuable tools to study P2X7R expression.

#### Reporter Mice Expressing EGFP-Tagged P2X7R (P2X7R-EGFP)

**FIGURE 2:**
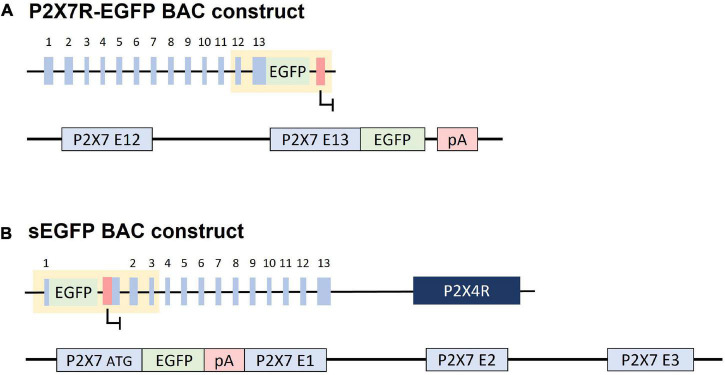
Constructs used for generation of BAC transgenic reporter mice. **(A)** P2X7R -EGFP: An EGFP cassette was inserted downstream of the P2X7R coding sequence in exon 13 creating a P2X7R -EGFP fusion transcript. **(B)** sEGFP: An EGFP cassette was inserted at the ATG of the P2X7R in exon 1, theoretically blocking the transcription of the remainder of the gene through its poly A signal. The *P2rx4* gene is present 16 kb downstream of the P2X7R construct. “pA/red box”: Polyadenylation signal.

The P2X7R -EGFP mouse line [Tg(RP24-114E20P2X7451P-Strep-His-EGFP)17Ani] is a bacterial artificial chromosomes (BAC) transgenic mouse line, generated by adding an EGFP sequence at the C-terminus of the P2X7R producing a P2X7R -EGFP fusion protein (Figure 2A). The resulting mouse was tested for receptor functionality as well as for its expression pattern. No significant difference in physiological responses between the native and the fusion protein were found, concluding that the EGFP-tag has no influence on P2X7R function (Kaczmarek-Hajek et al., 2018). However, Southern blot analysis shows that several copies of the P2X7R -EGFP construct are integrated into the genome of these mice, which means that this line is considerably overexpressing P2X7R (Kaczmarek-Hajek et al., 2018).

#### Reporter Mice Expressing Soluble EGFP

The soluble EGFP (sEGFP) mouse line [Tg(P2rx7-EGFP)FY174Gsat] is a BAC transgenic line generated by the Gene Expression Nervous System Atlas (GENSAT) project by insertion of an EGFP cassette followed by a polyA signal downstream of exon 1 of the *P2RX7* gene (Figure 2B). Therefore, EGFP expression is driven by the P2X7R promoter and supposed to mirror the endogenous P2X7R expression pattern. Different from the P2X7R -EGFP mouse line, the receptor cannot be detected directly, but only approximated based on the extent of the P2X7R promoter activity (Gong et al., 2003). The recent detailed characterization of this mouse line revealed that there is unexpected P2X7R and P2X4R overexpression. Moreover, these mice show an aberrant expression pattern compared to the endogenous P2X7R, questioning their use as a valid reporter line (Ramírez-Fernández et al., 2020).

## Discussion

The P2X7R is a relevant target for several disorders of the CNS due to its demonstrated involvement in response to cellular stress. It activates the production of inflammatory mediators, which have been associated with depression, suggesting the possibility that interfering with the physiology of P2X7R may have an impact on depression development and progression. Although P2X7R has been widely studied, there are still many unanswered questions related to it. First, there are studies that link certain P2X7R SNPs to depression, but the underlying consequences of these associations are still not fully understood. In fact, even the expression pattern of P2X7R has not been elucidated yet, and therefore, the physiological and pathophysiological pathways in which it is involved still need to be thoroughly analyzed.

Inactivating P2X7R seems to lead to altered anxiety- and depression-related phenotypes. However, it is now known that the knockout mouse models employed still have active P2X7R splice variants. Furthermore, some studies found significant behavioral effects of P2X7R inactivation under baseline conditions, while others only observed them under stress, or did not observe them at all. These facts make apparent the need for a reassessment of anxiety and depressive-like behaviors in a validated full P2X7R knockout mouse line. Overexpression of P2X7R is a phenomenon that has not been exploited in depression research. The P2X7R -EGFP mouse line presents P2X7R overexpression consistent with the endogenous pattern. Therefore, this mouse line could be useful to study the effect of P2X7R overexpression on various phenotypes.

Depression is a complex, multifactorial, heterogeneous disorder, with a relatively high ratio of treatment-resistance (McIntyre et al., 2014). Although it is known that anti-inflammatory mechanisms are involved in the treatment response of depression, current therapies are still mainly “one fits all” approaches targeting monoaminergic pathways (van Buel et al., 2015; Otte et al., 2016; Wohleb et al., 2016; Kruse et al., 2018; Binder, 2020; Nettis et al., 2021). To progress toward an effective application of P2X7R -targeting drugs in a clinical setting, there is a need to understand the involvement of P2X7R in psychiatric disorders (Deussing and Arzt, 2018). To that end, it is important to invest in reproducible studies that apply transgenic mouse models to offer novel perspectives on P2X7R signaling. Animal models allow the study of certain depression-related features, such as anxiety, anhedonia, or coping mechanisms. However, other aspects inherent to depression cannot be measured, such as guilt, thoughts of death or hopelessness. Behavioral tests and research frameworks need to evolve past current limitations toward comprehensive and standardized phenotyping reflective of human depression heterogeneity. Gradual and collaborative methodological refinement of behavioral and neurogenetic paradigms in psychiatry research will potentiate the value of animal-derived data in the context of depression and promote the translation of P2X7R evidence into clinical research.

## Author Contributions

LU-T reviewed the scientific literature and wrote the manuscript. I-AM-H contributed to revision and structuring of the manuscript. JD reviewed and edited the manuscript and provided scientific advice and guidance. All authors contributed to the article and approved the submitted version.

## Conflict of Interest

The authors declare that the research was conducted in the absence of any commercial or financial relationships that could be construed as a potential conflict of interest.

## Publisher’s Note

All claims expressed in this article are solely those of the authors and do not necessarily represent those of their affiliated organizations, or those of the publisher, the editors and the reviewers. Any product that may be evaluated in this article, or claim that may be made by its manufacturer, is not guaranteed or endorsed by the publisher.

## References

[B1] Abkevich V. Camp N. J. Hensel C. H. Neff C. D. Russell D. L. Hughes D. C. (2003). Predisposition locus for major depression at chromosome 12q22-12q23.2. *Am. J. Hum. Genet.* 73 1271–1281. 10.1086/379978 14606042 PMC1180393

[B2] Adinolfi E. Cirillo M. Woltersdorf R. Falzoni S. Chiozzi P. Pellegatti P. (2010). Trophic activity of a naturally occurring truncated isoform of the P2X7 receptor. *FASEB J.* 24 3393–3404. 10.1096/fj.09-153601 20453110

[B3] Adinolfi E. Giuliani A. L. De Marchi E. Pegoraro A. Orioli E. Di Virgilio F. (2018). The P2X7 receptor: a main player in inflammation. *Biochem. Pharmacol.* 151 234–244. 10.1016/j.bcp.2017.12.021 29288626

[B4] Anderson C. M. Nedergaard M. (2006). Emerging challenges of assigning P2X7 receptor function and immunoreactivity in neurons. *Trends Neurosci.* 29 257–262. 10.1016/j.tins.2006.03.003 16564580

[B5] Andrejew R. Oliveira-Giacomelli Á Ribeiro D. E. Glaser T. Arnaud-Sampaio V. F. Lameu C. (2020). The P2X7 receptor: central hub of brain diseases. *Front. Mol. Neurosci.* 13:124. 10.3389/fnmol.2020.00124 32848594 PMC7413029

[B6] Barden N. Harvey M. Gagné B. Shink E. Tremblay M. Raymond C. (2006). Analysis of single nucleotide polymorphisms in genes in the chromosome 12Q24.31 region points to P2RX7 as a susceptibility gene to bipolar affective disorder. *Am. J. Med. Genet. Part B Neuropsychiatr. Genet.* 141 374–382. 10.1002/ajmg.b.30303 16673375

[B7] Barnes J. Mondelli V. Pariante C. M. (2017). Genetic Contributions of Inflammation to Depression. *Neuropsychopharmacology* 42 81–98. 10.1038/npp.2016.169 27555379 PMC5143493

[B8] Bartlett R. Stokes L. Sluyter R. (2014). The P2X7 receptor channel: recent developments and the use of P2X7 antagonists in models of disease. *Pharmacol. Rev.* 66 638–675. 10.1124/pr.113.008003 24928329

[B9] Bartoli F. Burnstock G. Crocamo C. Carrà G. (2020). Purinergic signaling and related biomarkers in depression. *Brain Sci.* 10 1–12. 10.3390/brainsci10030160 32178222 PMC7139781

[B10] Basso A. M. Bratcher N. A. Harris R. R. Jarvis M. F. Decker M. W. Rueter L. E. (2009). Behavioral profile of P2X7 receptor knockout mice in animal models of depression and anxiety: relevance for neuropsychiatric disorders. *Behav. Brain Res.* 198 83–90. 10.1016/J.BBR.2008.10.018 18996151

[B11] Binder E. B. (2020). Understanding the mechanisms of treatment response in depression, focus on electro-convulsive therapy. *Eur. Arch. Psychiatry Clin. Neurosci.* 270 789–791. 10.1007/s00406-020-01184-1 32865635 PMC7474713

[B12] Boucher A. A. Arnold J. C. Hunt G. E. Spiro A. Spencer J. Brown C. (2011). Resilience and reduced c-Fos expression in P2X7 receptor knockout mice exposed to repeated forced swim test. *Neuroscience* 189 170–177. 10.1016/j.neuroscience.2011.05.049 21664437

[B13] Buch A. M. Liston C. (2021). Dissecting diagnostic heterogeneity in depression by integrating neuroimaging and genetics. *Neuropsychopharmacology* 46 156–175. 10.1038/s41386-020-00789-3 32781460 PMC7688954

[B14] Burnstock G. (2006). Purinergic signalling. *Br. J. Pharmacol.* 147 172–181. 10.1038/sj.bjp.0706429 16402102 PMC1760723

[B15] Calcia M. A. Bonsall D. R. Bloomfield P. S. Selvaraj S. Barichello T. Howes O. D. (2016). Stress and neuroinflammation: a systematic review of the effects of stress on microglia and the implications for mental illness. *Psychopharmacology* 233 1637–1650. 10.1007/s00213-016-4218-9 26847047 PMC4828495

[B16] Calzaferri F. Ruiz-Ruiz C. de Diego A. M. G. de Pascual R. Méndez-López I. Cano-Abad M. F. (2020). The purinergic P2X7 receptor as a potential drug target to combat neuroinflammation in neurodegenerative diseases. *Med. Res. Rev.* 40 2427–2465. 10.1002/med.21710 32677086

[B17] Cheewatrakoolpong B. Gilchrest H. Anthes J. C. Greenfeder S. (2005). Identification and characterization of splice variants of the human P2X7 ATP channel. *Biochem. Biophys. Res. Commun.* 332 17–27. 10.1016/j.bbrc.2005.04.087 15896293

[B18] Colonna M. Butovsky O. (2017). Microglia function in the central nervous system during health and neurodegeneration. *Annu. Rev. Immunol.* 35 441–468. 10.1146/annurev-immunol-051116-052358 28226226 PMC8167938

[B19] Csölle C. Andó R. D. Kittel Á Gölöncsér F. Baranyi M. Soproni K. (2013a). The absence of P2X7 receptors (P2rx7) on non-haematopoietic cells leads to selective alteration in mood-related behaviour with dysregulated gene expression and stress reactivity in mice. *Int. J. Neuropsychopharmacol.* 16 213–233. 10.1017/S1461145711001933 22243662 PMC3666310

[B20] Csölle C. Baranyi M. Zsilla G. Kittel Á Gölöncsér F. Illes P. (2013b). Neurochemical changes in the mouse hippocampus underlying the antidepressant effect of genetic deletion of P2X7 receptors. *PLoS One* 8:6547. 10.1371/journal.pone.0066547 23805233 PMC3689833

[B21] Cuijpers P. Stringaris A. Wolpert M. (2020). Treatment outcomes for depression: challenges and opportunities. *Lancet Psychiatry* 7 925–927. 10.1016/S2215-0366(20)30036-5 32078823

[B22] Curtis D. Kalsi G. Brynjolfsson J. McInnis M. O’Neill J. Smyth C. (2003). Genome scan of pedigrees multiply affected with bipolar disorder provides further support for the presence of a susceptibility locus on chromosome 12q23-q24, and suggests the presence of additional loci on 1p and 1q. *Psychiatr. Genet* 13 77–84. 10.1097/01.ypg.0000056684.89558.d2 12782963

[B23] Czamara D. Müller-Myhsok B. Lucae S. (2018). The P2RX7 polymorphism rs2230912 is associated with depression: a meta-analysis. *Prog. Neuro Psychopharmacol. Biol. Psychiatry* 82 272–277. 10.1016/J.PNPBP.2017.11.003 29122639

[B24] de Kluiver H. Jansen R. Milaneschi Y. Penninx B. W. J. H. (2019). Involvement of inflammatory gene expression pathways in depressed patients with hyperphagia. *Transl. Psychiatry* 9:193. 10.1038/s41398-019-0528-0 31431611 PMC6702221

[B25] de Kluiver H. Milaneschi Y. Jansen R. van Sprang E. D. Giltay E. J. Hartman C. A. (2021). Associations between depressive symptom profiles and immunometabolic characteristics in individuals with depression and their siblings. *World J. Biol. Psychiatry* 22 128–138. 10.1080/15622975.2020.1761562 32425087

[B26] Degn B. Lundorf M. D. Wang A. Vang M. Mors O. Kruse T. A. (2001). Further evidence for a bipolar risk gene on chromosome 12q24 suggested by investigation of haplotype sharing and allelic association in patients from the Faroe Islands. *Mol. Psychiatry* 6 450–455. 10.1038/sj.mp.4000882 11443532

[B27] Delic S. Streif S. Deussing J. M. Weber P. Ueffing M. Hölter S. M. (2008). Genetic mouse models for behavioral analysis through transgenic RNAi technology. *Genes Brain Behav.* 7 821–830. 10.1111/j.1601-183X.2008.00412.x 18518923

[B28] Deussing J. M. Arzt E. (2018). P2X7 receptor: a potential therapeutic target for depression? *Trends Mol. Med* 24 736–747. 10.1016/j.molmed.2018.07.005 30093269

[B29] Di Virgilio F. Dal Ben D. Sarti A. C. Giuliani A. L. Falzoni S. (2017). The P2X7 Receptor in Infection and Inflammation. *Immunity* 47 15–31. 10.1016/j.immuni.2017.06.020 28723547

[B30] Di Virgilio F. Schmalzing G. Markwardt F. (2018). The Elusive P2X7 Macropore. *Trends Cell Biol.* 28 392–404. 10.1016/j.tcb.2018.01.005 29439897

[B31] Donnelly-Roberts D. L. Namovic M. T. Han P. Jarvis M. F. (2009). Mammalian P2X7 receptor pharmacology: comparison of recombinant mouse, rat and human P2X7 receptors. *Br. J. Pharmacol.* 157 1203–1214. 10.1111/j.1476-5381.2009.00233.x 19558545 PMC2743839

[B32] Douguet L. Janho dit Hreich S. Benzaquen J. Seguin L. Juhel T. Dezitter X. (2021). A small-molecule P2RX7 activator promotes anti-tumor immune responses and sensitizes lung tumor to immunotherapy. *Nat. Commun.* 12:653. 10.1038/s41467-021-20912-2 33510147 PMC7843983

[B33] Draganov M. Arranz M. J. Salazar J. de Diego-Adeliño J. Gallego-Fabrega C. Jubero M. (2019). Association study of polymorphisms within inflammatory genes and methylation status in treatment response in major depression. *Eur. Psychiatry* 60 7–13. 10.1016/j.eurpsy.2019.05.003 31100612

[B34] Dubyak G. R. (2012). P2X7 receptor regulation of non-classical secretion from immune effector cells. *Cell. Microbiol.* 14 1697–1706. 10.1111/cmi.12001 22882764 PMC3473166

[B35] Feng W. P. Zhang B. Li W. Liu J. (2014). Lack of association of P2RX7 gene rs2230912 polymorphism with mood disorders: a meta-analysis. *PLoS One* 9:e88575. 10.1371/journal.pone.0088575 24533115 PMC3922924

[B36] Feng Y.-H. Li X. Wang L. Zhou L. Gorodeski G. I. (2006). A truncated P2X7 receptor variant (P2X7-j) endogenously expressed in cervical cancer cells antagonizes the full-length P2X7 receptor through hetero-oligomerization. *J. Biol. Chem.* 281 17228–17237. 10.1074/jbc.M602999200 16624800 PMC2409001

[B37] Friedrich M. J. (2017). Depression is the leading cause of disability around the world. *JAMA* 317:1517. 10.4337/9781783478651 28418490

[B38] Gao L. Lin Z. Xie G. Zhou T. Hu W. Liu C. (2018). The effects of P2X7 receptor knockout on emotional conditions over the lifespan of mice. *Neuroreport* 29 1479–1486. 10.1097/WNR.0000000000001136 30281537

[B39] Gilman S. E. Sucha E. Kingsbury M. Horton N. J. Murphy J. M. Colman I. (2017). Depression and mortality in a longitudinal study: 1952-2011. *CMAJ* 189 E1304–E1310. 10.1503/cmaj.170125 29061855 PMC5654987

[B40] Giollabhui N. Mac Ng T. Ellman L. M. Alloy L. B. (2020). The longitudinal associations of inflammatory biomarkers and depression revisited: systematic review. Meta-Analysis and Meta-Regression. *Biol. Psychiatry* 87:S450. 10.1016/j.biopsych.2020.02.1146 PMC7887136 32807846

[B41] Gong S. Zheng C. Doughty M. L. Losos K. Didkovsky N. Schambra U. B. (2003). A gene expression atlas of the central nervous system based on bacterial artificial chromosomes. *AAPM Annu. Meet.* 425 917–925. 10.1038/nature02033 14586460

[B42] Green E. K. Grozeva D. Raybould R. Elvidge G. Macgregor S. Craig I. (2009). P2RX7: a bipolar and unipolar disorder candidate susceptibility gene? *Am. J. Med. Genet. Part B Neuropsychiatr. Genet.* 150 1063–1069. 10.1002/ajmg.b.30931 19160446

[B43] Grigoroiu-Serbanescu M. Herms S. Mühleisen T. W. Georgi A. Diaconu C. C. Strohmaier J. (2009). Variation in P2RX7 candidate gene (rs2230912) is not associated with bipolar I disorder and unipolar major depression in four European samples. *Am. J. Med. Genet. Part B Neuropsychiatr. Genet.* 150B 1017–1021. 10.1002/ajmg.b.30952 19330776

[B44] Habermacher C. Dunning K. Chataigneau T. Grutter T. (2016). Molecular structure and function of P2X receptors. *Neuropharmacology* 104 18–30. 10.1016/j.neuropharm.2015.07.032 26231831

[B45] Hattori M. Gouaux E. (2012). Molecular mechanism of ATP binding and ion channel activation in P2X receptors. *Nature* 485 207–212. 10.1038/nature11010 22535247 PMC3391165

[B46] Hejjas K. Szekely A. Domotor E. Halmai Z. Balogh G. Schilling B. (2009). Association between depression and the Gln460Arg polymorphism of P2RX7 gene: a dimensional approach. *Am. J. Med. Genet. Part B Neuropsychiatr. Genet.* 150 295–299. 10.1002/ajmg.b.30799 18543274

[B47] Illes P. Khan T. M. Rubini P. (2017). Neuronal P2X7 receptors revisited: do they really exist? *J. Neurosci.* 37 7049–7062. 10.1523/JNEUROSCI.3103-16.2017 28747388 PMC6705732

[B48] Illes P. Verkhratsky A. Tang Y. (2020). Pathological ATPergic signaling in major depression and bipolar disorder. *Front. Mol. Neurosci.* 12:331. 10.3389/fnmol.2019.00331 32076399 PMC7006450

[B49] Iwata M. Ota K. T. Duman R. S. (2013). The inflammasome: pathways linking psychological stress, depression, and systemic illnesses. *Brain. Behav. Immun.* 31 105–114. 10.1016/j.bbi.2012.12.008 23261775 PMC4426992

[B50] Iwata M. Ota K. T. Li X.-Y. Y. Sakaue F. Li N. Dutheil S. (2016). Psychological stress activates the inflammasome via release of adenosine triphosphate and stimulation of the purinergic type 2X7 receptor. *Biol. Psychiatry* 80 12–22. 10.1016/j.biopsych.2015.11.026 26831917

[B51] Jacobson K. A. Müller C. E. (2016). Medicinal chemistry of adenosine, P2Y and P2X receptors. *Neuropharmacology* 104 31–49. 10.1016/j.neuropharm.2015.12.001 26686393 PMC4871727

[B52] James S. L. Abate D. Abate K. H. Abay S. M. Abbafati C. Abbasi N. (2018). Global, regional, and national incidence, prevalence, and years lived with disability for 354 Diseases and Injuries for 195 countries and territories, 1990-2017: a systematic analysis for the Global Burden of Disease Study 2017. *Lancet* 392 1789–1858. 10.1016/S0140-6736(18)32279-7 30496104 PMC6227754

[B53] Kaczmarek-Hajek K. Zhang J. Kopp R. Grosche A. Rissiek B. Saul A. (2018). Re-evaluation of neuronal P2X7 expression using novel mouse models and a P2X7-specific nanobody. *Elife* 7:e36217. 10.7554/eLife.36217 30074479 PMC6140716

[B54] Kappelmann N. Arloth J. Georgakis M. K. Czamara D. Rost N. Ligthart S. (2021). Dissecting the association between inflammation. Metabolic dysregulation, and specific depressive symptoms: a genetic correlation and 2-sample mendelian randomization study. *JAMA Psychiatry* 78 161–170. 10.1001/jamapsychiatry.2020.3436 33079133 PMC7577200

[B55] Karasawa A. Kawate T. (2016). Structural basis for subtype-specific inhibition of the P2X7 receptor. *Elife* 5:e22153. 10.7554/eLife.22153 27935479 PMC5176352

[B56] Kendler K. S. Karkowski L. M. Prescott C. A. (1999). Causal relationship between stressful life events and the onset of major depression. *Am. J. Psychiatry* 156 837–841. 10.1176/ajp.156.6.837 10360120

[B57] Kessler R. C. (2012). The costs of depression. *Psychiatr. Clin. North Am.* 35 1–14. 10.1016/j.psc.2011.11.005 22370487 PMC3292769

[B58] Khadra A. Tomić M. Yan Z. Zemkova H. Sherman A. Stojilkovic S. S. (2013). Dual gating mechanism and function of P2X7 receptor channels. *Biophys. J.* 104 2612–2621. 10.1016/j.bpj.2013.05.006 23790369 PMC3686336

[B59] Khakh B. S. North R. A. (2012). Neuromodulation by extracellular ATP and P2X receptors in the CNS. *Neuron* 76 51–69. 10.1016/j.neuron.2012.09.024 23040806 PMC4064466

[B60] Kim J. Yoon S. Lee S. Hong H. Ha E. Joo Y. (2020). A double-hit of stress and low-grade inflammation on functional brain network mediates posttraumatic stress symptoms. *Nat. Commun.* 11:1898. 10.1038/s41467-020-15655-5 32313055 PMC7171097

[B61] Kruse J. L. Congdon E. Olmstead R. Njau S. Breen E. Narr K. L. (2018). Inflammation and improvement of depression following electroconvulsive therapy in treatment-resistant depression. *J. Clin. Psychiatry* 79:17m11597. 10.4088/JCP.17m11597 29489077 PMC6013272

[B62] Leday G. G. R. Vértes P. E. Richardson S. Greene J. R. Regan T. Khan S. (2018). Replicable and coupled changes in innate and adaptive immune gene expression in two case-control studies of blood microarrays in major depressive disorder. *Biol. Psychiatry* 83 70–80. 10.1016/j.biopsych.2017.01.021 28688579 PMC5720346

[B63] Lim G. Y. Tam W. W. Lu Y. Ho C. S. Zhang M. W. Ho R. C. (2018). Prevalence of depression in the community from 30 countries between 1994 and 2014/692/699/476/1414/692/499 article. *Sci. Rep.* 8:2861. 10.1038/s41598-018-21243-x 29434331 PMC5809481

[B64] Liu Q. He H. Yang J. Feng X. Zhao F. Lyu J. (2020). Changes in the global burden of depression from 1990 to 2017: findings from the global burden of disease study. *J. Psychiatr. Res.* 126 134–140. 10.1016/j.jpsychires.2019.08.002 31439359

[B65] Lucae S. Salyakina D. Barden N. Harvey M. Gagné B. Labbé M. (2006). P2RX7, a gene coding for a purinergic ligand-gated ion channel, is associated with major depressive disorder. *Hum. Mol. Genet.* 15 2438–2445. 10.1093/hmg/ddl166 16822851

[B66] Machado M. O. Veronese N. Sanches M. Stubbs B. Koyanagi A. Thompson T. (2018). The association of depression and all-cause and cause-specific mortality: an umbrella review of systematic reviews and meta-analyses. *BMC Med.* 16:112. 10.1186/s12916-018-1101-z 30025524 PMC6053830

[B67] Masin M. Young C. Lim K. Barnes S. J. Xu X. J. Marschall V. (2012). Expression, assembly and function of novel C-terminal truncated variants of the mouse P2X7 receptor: re-evaluation of P2X7 knockouts. *Br. J. Pharmacol.* 165 978–993. 10.1111/J.1476-5381.2011.01624.X 21838754 PMC3312493

[B68] Maydych V. (2019). The interplay between stress, inflammation, and emotional attention: relevance for depression. *Front. Neurosci.* 13:384. 10.3389/fnins.2019.00384 31068783 PMC6491771

[B69] McCarthy A. E. Yoshioka C. Mansoor S. E. (2019). Full-Length P2X7 structures reveal how palmitoylation prevents channel desensitization. *Cell* 179 659–670.e13. 10.1016/j.cell.2019.09.017 31587896 PMC7053488

[B70] McGuffin P. Knight J. Breen G. Brewster S. Boyd P. R. Craddock N. (2005). Whole genome linkage scan of recurrent depressive disorder from the depression network study. *Hum. Mol. Genet.* 14 3337–3345. 10.1093/hmg/ddi363 16203746

[B71] McIntyre R. S. Filteau M. Martin L. Patry S. Carvalho A. Cha D. S. (2014). Treatment-resistant depression: definitions, review of the evidence, and algorithmic approach. *J. Affect. Disord* 156 1–7. 10.1016/j.jad.2013.10.043 24314926

[B72] Medina-Rodriguez E. M. Lowell J. A. Worthen R. J. Syed S. A. Beurel E. (2018). Involvement of innate and adaptive immune systems alterations in the pathophysiology and treatment of depression. *Front. Neurosci.* 12:547. 10.3389/fnins.2018.00547 30174579 PMC6107705

[B73] Metzger M. W. Walser S. M. Dedic N. Aprile-Garcia F. Jakubcakova V. Adamczyk M. (2017b). Heterozygosity for the mood disorder-associated variant Gln460Arg alters P2X7 receptor function and sleep quality. *J. Neurosci.* 37 11688–11700. 10.1523/JNEUROSCI.3487-16.2017 29079688 PMC6705750

[B74] Metzger M. W. Walser S. M. Aprile-Garcia F. Dedic N. Chen A. Holsboer F. (2017a). Genetically dissecting P2rx7 expression within the central nervous system using conditional humanized mice. *Purinergic Signal.* 13 153–170. 10.1007/s11302-016-9546-z 27858314 PMC5432476

[B75] Milaneschi Y. Lamers F. Berk M. Penninx B. W. J. H. (2020). Depression heterogeneity and its biological underpinnings: toward immunometabolic depression. *Biol. Psychiatry* 88 369–380. 10.1016/j.biopsych.2020.01.014 32247527

[B76] Monif M. Reid C. A. Powell K. L. Drummond K. J. O’Brien T. J. Williams D. A. (2016). Interleukin-1β has trophic effects in microglia and its release is mediated by P2X7R pore. *J. Neuroinflammation* 13 1–15. 10.1186/s12974-016-0621-8 27364756 PMC4929731

[B77] Moore S. F. MacKenzie A. B. (2008). Species and agonist dependent zinc modulation of endogenous and recombinant ATP-gated P2X7 receptors. *Biochem. Pharmacol.* 76 1740–1747. 10.1016/j.bcp.2008.09.015 18848528

[B78] Musil R. Seemüller F. Meyer S. Spellmann I. Adli M. Bauer M. (2018). Subtypes of depression and their overlap in a naturalistic inpatient sample of major depressive disorder. *Int. J. Methods Psychiatr. Res.* 27 1–16. 10.1002/mpr.1569 29498147 PMC6877097

[B79] Nagy G. Ronai Z. Somogyi A. Sasvari-Szekely M. Rahman O. A. Mate A. (2008). P2RX7 Gln460Arg polymorphism is associated with depression among diabetic patients. *Prog. Neuro Psychopharmacol. Biol. Psychiatry* 32 1884–1888. 10.1016/j.pnpbp.2008.08.021 18801407

[B80] Nelson J. Klumparendt A. Doebler P. Ehring T. (2017). Childhood maltreatment and characteristics of adult depression: meta-analysis. *Br. J. Psychiatry* 210 96–104. 10.1192/bjp.bp.115.180752 27908895

[B81] Nettis M. A. Lombardo G. Hastings C. Zajkowska Z. Mariani N. Nikkheslat N. (2021). Augmentation therapy with minocycline in treatment-resistant depression patients with low-grade peripheral inflammation: results from a double-blind randomised clinical trial. *Neuropsychopharmacology* 46 939–948. 10.1038/s41386-020-00948-6 33504955 PMC8096832

[B82] Nguyen T. D. Hieronymus F. Lorentzen R. McGirr A. Østergaard S. D. (2021). The efficacy of repetitive transcranial magnetic stimulation (rTMS) for bipolar depression: a systematic review and meta-analysis. *J. Affect. Disord.* 279 250–255. 10.1016/j.jad.2020.10.013 33074144

[B83] Nicke A. Kuan Y.-H. Masin M. Rettinger J. Marquez-Klaka B. Bender O. (2009). A functional P2X7 splice variant with an alternative transmembrane domain 1 escapes gene inactivation in P2X7 knock-out mice. *J. Biol. Chem.* 284 25813–25822. 10.1074/jbc.M109.033134 19546214 PMC2757983

[B84] Otte C. Gold S. M. Penninx B. W. Pariante C. M. Etkin A. Fava M. (2016). Major depressive disorder. *Nat. Rev. Dis. Primers.* 2:16065. 10.1038/nrdp.2016.65 27629598

[B85] Pegoraro A. De Marchi E. Adinolfi E. (2021). P2X7 Variants in Oncogenesis. *Cells* 10:189. 10.3390/cells10010189 33477845 PMC7832898

[B86] Peterson R. E. Cai N. Dahl A. W. Bigdeli T. B. Edwards A. C. Webb B. T. (2018). Molecular genetic analysis subdivided by adversity exposure suggests etiologic heterogeneity in major depression. *Am. J. Psychiatry* 175 545–554. 10.1176/appi.ajp.2017.17060621 29495898 PMC5988935

[B87] Ramírez-Fernández A. Urbina-Treviño L. Conte G. Alves M. Rissiek B. Durner A. (2020). Deviant reporter expression and P2X4 passenger gene overexpression in the soluble EGFP BAC transgenic P2X7 reporter mouse model. *Sci. Rep.* 10:19876. 10.1038/s41598-020-76428-0 33199725 PMC7669894

[B88] Ribeiro D. E. Roncalho A. L. Glaser T. Ulrich H. Wegener G. Joca S. (2019). P2X7 receptor signaling in stress and depression. *Int. J. Mol. Sci.* 20 1–26. 10.3390/ijms20112778 31174279 PMC6600521

[B89] Rohleder N. (2014). Stimulation of systemic low-grade inflammation by psychosocial stress. *Psychosom. Med.* 76 181–189. 10.1097/PSY.0000000000000049 24608036

[B90] Schiweck C. Claes S. Van Oudenhove L. Lafit G. Vaessen T. de Beeck G. O. (2020). Childhood trauma, suicide risk and inflammatory phenotypes of depression: insights from monocyte gene expression. *Transl. Psychiatry* 10:296. 10.1038/s41398-020-00979-z 32839428 PMC7445278

[B91] Shink E. Morissette J. Sherrington R. Barden N. (2005). A genome-wide scan points to a susceptibility locus for bipolar disorder on chromosome 12. *Mol. Psychiatry* 10 545–552. 10.1038/sj.mp.4001601 15494705

[B92] Sikora A. Liu J. Brosnan C. Buell G. Chessel I. Bloom B. R. (1999). Cutting edge: purinergic signaling regulates radical-mediated bacterial killing mechanisms in macrophages through a P2X7-independent mechanism. *J. Immunol.* 163 558–561. 10395640

[B93] Sluyter R. (2017). The P2X7 receptor. *Adv. Exp. Med. Biol.* 1051 17–53. 10.1007/5584_2017_59 28676924

[B94] Sluyter R. Stokes L. (2011). Significance of P2X7 receptor variants to human health and disease. *Recent Pat. DNA Gene Seq.* 5 41–54. 10.2174/187221511794839219 21303345

[B95] Solle M. Labasi J. Perregaux D. G. Stam E. Petrushova N. Koller B. H. (2001). Altered cytokine production in mice lacking P2X(7) receptors. *J. Biol. Chem.* 276 125–132. 10.1074/jbc.M006781200 11016935

[B96] Sontheimer N. Konnopka A. König H. H. (2021). The excess costs of dementia: a systematic review and meta-analysis. *J. Alzheimers Dis.* 83 333–354. 10.3233/JAD-210174 34334395

[B97] Soronen P. Mantere O. Melartin T. Suominen K. Vuorilehto M. Rytsälä H. (2011). P2RX7 gene is associated consistently with mood disorders and predicts clinical outcome in three clinical cohorts. *Am. J. Med. Genet. Part B Neuropsychiatr. Genet.* 156 435–447. 10.1002/ajmg.b.31179 21438144

[B98] Stahl E. A. Breen G. Forstner A. J. McQuillin A. Ripke S. Trubetskoy V. (2019). Genome-wide association study identifies 30 loci associated with bipolar disorder. *Nat. Genet.* 51 793–803. 10.1038/s41588-019-0397-8 31043756 PMC6956732

[B99] Steffen A. Nübel J. Jacobi F. Bätzing J. Holstiege J. (2020a). Mental and somatic comorbidity of depression: a comprehensive cross-sectional analysis of 202 diagnosis groups using German nationwide ambulatory claims data. *BMC Psychiatry* 20:142. 10.1186/s12888-020-02546-8 32228541 PMC7106695

[B100] Steffen A. Thom J. Jacobi F. Holstiege J. Bätzing J. (2020b). Trends in prevalence of depression in Germany between 2009 and 2017 based on nationwide ambulatory claims data. *J. Affect. Disord.* 271 239–247. 10.1016/j.jad.2020.03.082 32479322

[B101] Stokes L. Fuller S. J. Sluyter R. Skarratt K. K. Gu B. J. Wiley J. S. (2010). Two haplotypes of the P2X 7 receptor containing the Ala-348 to Thr polymorphism exhibit a gain-of-function effect and enhanced interleukin-1β secretion. *FASEB J.* 24 2916–2927. 10.1096/fj.09-150862 20360457

[B102] Tokuda T. Yoshimoto J. Shimizu Y. Okada G. Takamura M. Okamoto Y. (2018). Identification of depression subtypes and relevant brain regions using a data-driven approach. *Sci. Rep.* 8:14082. 10.1038/s41598-018-32521-z 30237567 PMC6148252

[B103] Troubat R. Barone P. Leman S. Desmidt T. Cressant A. Atanasova B. (2020). Neuroinflammation and depression: a review. *Eur. J. Neurosci.* 53 151–171. 10.1111/ejn.14720 32150310

[B104] van Buel E. M. Patas K. Peters M. Bosker F. J. Eisel U. L. M. Klein H. C. (2015). Immune and neorutrophin stimulation by electroconvulsive therapy: is some inflammation needed after all? *Transl. Psychiatry.* 5:e609. 10.1038/tp.2015.100 26218851 PMC5068722

[B105] Vereczkei A. Abdul-Rahman O. Halmai Z. Nagy G. Szekely A. Somogyi A. (2019). Association of purinergic receptor P2RX7 gene polymorphisms with depression symptoms. *Prog. Neuro Psychopharmacol. Biol. Psychiatry* 92 207–216. 10.1016/j.pnpbp.2019.01.006 30664971

[B106] Verkhratsky A. Burnstock G. (2014). Biology of purinergic signalling: its ancient evolutionary roots, its omnipresence and its multiple functional significance. *Bioessays* 36 697–705. 10.1002/bies.201400024 24782352

[B107] Viikki M. Kampman O. Anttila S. Illi A. Setälä-Soikkeli E. Huuhka M. (2011). P2RX7 polymorphisms Gln460Arg and His155Tyr are not associated with major depressive disorder or remission after SSRI or ECT. *Neurosci. Lett.* 493 127–130. 10.1016/j.neulet.2011.02.023 21335057

[B108] von Muecke-Heim I. A. Ries C. Urbina L. Deussing J. M. (2021). P2X7R antagonists in chronic stress-based depression models: a review. *Eur. Arch. Psychiatry Clin. Neurosci.* 271 1343–1358. 10.1007/s00406-021-01306-3 34279714 PMC8429152

[B109] Wohleb E. S. Franklin T. Iwata M. Duman R. S. (2016). Integrating neuroimmune systems in the neurobiology of depression. *Nat. Rev. Neurosci.* 17 497–511. 10.1038/nrn.2016.69 27277867

[B110] World Health Organizaton [WHO] (2017). *Depression and Other Common Mental Disorders: Global Health Estimates.* Geneva: World Health Organization.

[B111] Wray N. R. Ripke S. Mattheisen M. Trzaskowski M. Byrne E. M. Abdellaoui A. (2018). Genome-wide association analyses identify 44 risk variants and refine the genetic architecture of major depression. *Nat. Genet.* 50 668–681. 10.1038/s41588-018-0090-3 29700475 PMC5934326

[B112] Yue N. Huang H. Zhu X. Han Q. Wang Y. Li B. (2017). Activation of P2X7 receptor and NLRP3 inflammasome assembly in hippocampal glial cells mediates chronic stress-induced depressive-like behaviors. *J. Neuroinflammation* 14 1–15. 10.1186/s12974-017-0865-y 28486969 PMC5424302

